# The waist-to-height ratio is a good predictor for insulin resistance in women with polycystic ovary syndrome

**DOI:** 10.3389/fendo.2024.1502321

**Published:** 2024-12-09

**Authors:** Mengyi Zhu, Kaiyue Wang, Jiaxing Feng, Yang Liu, Muxin Guan, Yu Wang, Xiaoke Wu

**Affiliations:** ^1^ Graduate School, Heilongjiang University of Chinese Medicine, Harbin, China; ^2^ First Affiliated Hospital, Heilongjiang University of Chinese Medicine, Harbin, China

**Keywords:** anthropometric measurements, insulin resistance, polycystic ovary syndrome, prediction, waist-to-height ratio

## Abstract

**Objective:**

This study aimed to explore the role of the waist-to-height ratio (WHtR) in assessing insulin resistance (IR) in patients with polycystic ovary syndrome (PCOS).

**Materials and methods:**

We enrolled 882 PCOS-afflicted women in a cross-sectional analysis to evaluate the association of the WHtR with IR. Their demographic characteristics, anthropometric parameters, and fasting blood samples were collected and measured. Moreover, IR was evaluated by homeostatic model assessment of insulin resistance (HOMA-IR). We estimated the relationship between the WHtR and IR and the cut-off thresholds of the WHtR for IR using multivariable linear regression and logistic regression models, respectively.

**Result(s):**

The prevalence rate of IR was 51.9%. The patients with PCOS and IR displayed significantly increased values for body mass index (BMI), waist circumference (WC), WHtR, systolic blood pressure (SBP), diastolic blood pressure (DBP), free androgen index (FAI), HOMA-IR, total cholesterol (TC), triglyceride (TG), low-density lipoprotein cholesterol (LDL-C), and apolipoprotein B (ApoB). However, the patients with PCOS and IR showed a reduction in estradiol (E2), luteinizing hormone (LH), LH/FSH ratio, sex hormone binding globulin (SHBG), and high-density lipoprotein (HDL-C) values than those without IR. Moreover, BMI (log-transformed), WC, and HOMA-IR (log-transformed) were positively correlated with the WHtR. When adjusting for potential confounding variables, the WHtR was significantly associated with HOMA-IR (log-transformed), with a standardized regression coefficient of 0.271. Furthermore, the WHtR was significantly associated with an increased risk of IR, with the adjusted odds ratio (OR) of 3.15 (WHtR multiplied by 10). Additionally, the WHtR helped to identify IR in women with PCOS with an optimal cut-off point of 0.519 (Youden index = 0.433).

**Conclusion(s):**

The WHtR had a positive association with IR in women with PCOS. Hence, we suggest that the WHtR, as a simple, practical, and reliable anthropometric measure, can be used to predict the risk of IR in patients with PCOS.

## 1 Introduction

With a global prevalence ranging from 4% to 21%, polycystic ovary syndrome (PCOS) is one of the most frequent endocrine conditions affecting women of childbearing age (1). PCOS is also significantly associated with several endocrine and metabolic abnormalities, including insulin resistance (IR), cardiovascular risks, and androgen excess (2–4). Since IR has been identified as a pivotal pathogenic component of many metabolic diseases, early detection of IR in women with PCOS is crucial (5).

IR refers to an inadequate cellular response to insulin action, which upsets glucose homeostasis and leads to compensatory hyperinsulinemia, impaired fasting glucose, impaired glucose tolerance, or type 2 diabetes mellitus (6). Furthermore, IR is a disordered physiological response to insulin stimulation through interference in different molecular pathways in target tissues (7). For women with PCOS, IR could also selectively affect certain tissues’ metabolic or mitotic pathways, including their ovaries (8). IR is prevalent in PCOS-afflicted women and affects approximately 12% to 60% of them (9). Thus, IR could be a beneficial measure for managing PCOS and its associated complications (10). Since the hyperinsulinemic-euglycemic clamp, the “gold standard” detection method for IR, is complicated, additional methods and techniques to detect IR are now being explored (11). Common clinical detection techniques include direct and indirect methods such as the hyperinsulinemic-euglycemic clamp technique, the Quantitative Insulin Sensitivity Check Index (QUICKI), and the homeostasis model assessment of insulin resistance (HOMA-IR). However, all these methods require specialized equipment or trained professionals, which might make it more difficult to identify IR early and manage PCOS-related metabolic conditions.

Various anthropometric measurements, such as body mass index (BMI), waist-to-height ratio (WHtR), and waist circumference (WC), can help identify types or the status of abnormal metabolism (12–14). Moreover, patients with PCOS displayed more severe IR and sex-hormone disorders; negative correlations were observed between body fat and sex hormones in patients with PCOS compared with those without PCOS (15). This suggested that body fat assessment should be more specific in PCOS management. A simple and time-saving anthropometric measurement, the WHtR refers to central obesity status (16). It has been identified as a cardiovascular disease predictor (17, 18). A population-based cross-sectional study revealed that the WHtR is closely related to diabetes, especially in women (19). Another WHtR analysis suggested that it could be a better index of prevalent cardiac events than BMI, waist-to-hip ratio (WHR), and WC (20, 21). However, there are an insufficient number of studies on WHtR and PCOS to date. A 2016 study involving a cohort of 704 eumenorrheic non-hirsute subjects and 50 women with PCOS indicated that the WHtR can reliably predict IR and metabolic syndrome in PCOS-afflicted women. This finding suggests that the WHtR might become a promising PCOS screening tool (22). Recently, the WHtR has been suggested as a representative marker for metabolic syndrome assessment in PCOS cases (23). Additionally, a cross-sectional study on 66 Indian patients with PCOS suggested that the WHtR might be a beneficial screening tool for detecting IR early in the PCOS population (24).

Hence, we performed a cross-sectional study based on a large-sample randomized controlled trial and aimed to explore the relationship between the WHtR and IR in patients with PCOS by examining the association between the WHtR and HOMA-IR. We also sought to test the accuracy and reliability of the WHtR as a marker for IR in patients with PCOS.

## 2 Materials and methods

### 2.1 Participants

This was an observational study on the association between the WHtR and metabolic dysfunction in PCOS-afflicted women. We collected data from the Polycystic Ovary Syndrome Acupuncture plus Clomiphene Trial (PCOSAct), a large-sample, randomized controlled trial in China (25). The trial was registered on ClinicalTrials.gov (NCT01573858) and chictr.org.cn (ChiCTR-TRC-12002081). The key findings (25) and protocol (25) have been published.

The inclusion criteria were: Women with PCOS who were anovulatory and were trying ovulation induction. PCOS was defined by the modified Rotterdam criteria (26). The exclusion criteria were: Women with other endocrine disorders [including Hyperprolactinemia (defined as two prolactin levels that were at least one week apart >=25 ng/mL); those with FSH levels > 15 mIU/mL; uncorrected thyroid disease patients (defined as TSH <0.2 mIU/mL or >5.5 mIU/mL); those with poorly controlled Type I or Type II diabetes (HbA1c level > 7.0%) or patients on antidiabetic medications such as metformin, insulin, thiazolidinediones, acarbose, or sulfonylureas; and those with suspected Cushing syndrome]; Patients who used hormonal or other medications including Chinese herbal prescriptions in the past 2 months; those with a miscarriage or who had given birth within 6 weeks or who were breastfeeding within the last 6 months; and those without glucose or lipid measurements were excluded. Thus, 882 PCOSAct participants were eligible for the secondary analysis. All procedures followed the ethical standards of the institutional and/or national research committees and the principles of the Declaration of Helsinki and its subsequent amendments or comparable ethical standards.

### 2.2 Anthropometric and laboratory measurements

The evaluation included collecting patients’ demographic profiles, anthropometric parameters, and fasting blood samples. The demographic status refers to patients’ ages. The anthropometric measurements included height, weight, blood pressure, and WC. BMI was calculated as weight (kg) divided by the square of height (m) and the WHtR was calculated as WC (cm)/height (cm). WHR was calculated as WC (cm)/hip circumference (cm). We used patients from 21 PCOSAct centers. All research assistants underwent unified training at the project’s initial stage, covering measurement techniques for height, weight, WC, hip circumference, systolic blood pressure (SBP), and diastolic blood pressure (DBP), as well as blood sample collection and storage techniques.

The specific measurement methods were as follows. (1) Height (cm) was measured with the patient barefoot, eyes looking straight ahead, arms at their sides, and their back touching a ruler. Subsequently, a horizontal plate was pressed against the head to obtain an accurate reading to the nearest 0.1 cm measurement. (2) Weight (kg) was measured after the patients removed their hats, emptied their bladders and bowels, and wore lightweight clothing. Its accuracy was ensured to the nearest 0.1 kg measurement. (3) WC (cm) was measured with an empty bladder and bowels, with arms at their sides, and normal breathing. We used a tape measure around the navel, keeping it at the central point, and measuring to the nearest 0.1 cm. (4) Hip circumference (cm) was measured with an empty bladder and bowels with arms sideways and normal breathing. A tape measure was used to measure the horizontal circumference at the widest point of the hips to the nearest 0.1 cm. (5) Blood pressure (mmHg) was measured using a mercury sphygmomanometer. After resting quietly for approximately 10 minutes, the patients sat with their upper arms exposed and their elbows positioned at the same level as their hearts. The cuff was wrapped around the upper arm and positioned 2-3 cm above the antecubital fossa. A stethoscope was placed over the brachial artery. Both SBP and DBP were measured to the nearest 1 mmHg level.

The blood collection and storage standards were as follows. We collected 20 ml of whole blood from each subject at baseline. Of this, 10 ml were placed into an EDTA anticoagulant tube and aliquoted into cryovials. We placed the other 10 ml into a clot-activating tube, followed by serum separation and aliquoting into Eppendorf (EP) tubes in a refrigerator at -20°C or -80°C. Each subject’s blood samples were independently placed in a self-sealing bag, with a blood sample storage registration card indicating the specimen information, subject’s identification number, and sampling time. Subsequently, the samples were sent to the central laboratory in batches through a cold chain company for storage at -80°C. All biomarker tests were uniformly conducted at the Radioimmunoassay Special Examination Department of Heilongjiang Provincial Hospital and the Clinical Laboratory of the First Affiliated Hospital of Heilongjiang University of Chinese Medicine to minimize potential instrument errors.

Baseline fasting circulating glucose, lipid metabolism, sex steroids, and gonadotropin levels were measured at the core laboratory in the Heilongjiang University of Chinese Medicine. This included fasting glucose, total fasting insulin, total cholesterol (TC), triglyceride (TG), lipoprotein A, high-density lipoprotein cholesterol (HDL-C), low-density lipoprotein cholesterol (LDL-C), apolipoprotein A1 (ApoA1), apolipoprotein B (ApoB), total testosterone (TT), free testosterone (FT), sex hormone binding globulin (SHBG), estradiol (E2), progesterone (P), luteinizing hormone (LH), and follicle-stimulating hormone (FSH) levels. Moreover, glucose and lipid profiles were measured by enzymatic methods using a Hitachi Biochemical Analyzer (LABOSPE CT008). All sex hormones were analyzed using a Roche Cobas 6000-E601, an electrochemiluminescent immunoassay, except FT, which was measured using a radioimmunoassay (RIA). All intra-and inter-assay coefficient variation assessments were <5% and ≤10%, respectively. The HOMA-IR levels (fasting insulin[mIU/mL]×fasting glucose[mmol/L])/22.5), free androgen index (FAI = TT (nmol/L)/[SHBG(nmol/L)]×100), and LH to FSH (LH/FSH) ratio were calculated. Participants were considered to have IR when their HOMA-IR index was R≥2.6×10^-6^ mol×U/L^2^.

### 2.3 Statistical analyses

All statistical analyses were done using SPSS version 25.0 software (IBM Corporation). Data were described as the mean ± standard deviation or as the median (interquartile range), or number and percentage for continuous and categorical variables, respectively. Shapiro-Wilk tests suggested that BMI, WHR, SBP, DBP, E2, P, LH, LH/FSH ratio, FAI, SHBG, fasting glucose, fasting insulin, HOMA-IR, TG, and lipoprotein A were not normally distributed. Differences among the two groups were analyzed for continuous variables using Student’s t-test and the Mann-Whitney U test for those with normal and skewed distributions, respectively. Moreover, BMI and HOMA-IR were log-transformed before Pearson’s correlation analysis was performed. Pearson’s correlation analysis helped us to assess the correlation between the WHtR and BMI (log-transformed), WC, WHR, and HOMA-IR (log-transformed). Univariate linear regression analysis determined the association between HOMA-IR (log-transformed) and the WHtR. Variables with a *p*<0.05 were included in the multivariate linear regression and multivariable logistic regression analyses, respectively. We used the multivariable linear regression to explore the association of WHtR with HOMA-IR (log-transformed). Furthermore, multivariable logistic regression analysis helped us to calculate the adjusted odds ratios (OR) and 95% confidence interval (CI) of WHtR for IR in different models after potential confounder adjustment. Due to the excessively large OR value observed for the WHtR in the IR logistic regression analysis, the WHtR values were multiplied by 10 to achieve precise and interpretable OR values. In the multivariable linear regression and logistic regression analyses, we did not adjust the variables in model 1. However, age, SBP, and DBP were adjusted for in model 2, and E2, LH, LH/FSH ratio, FAI, SHBG, HDL-C, LDL-C, cholesterol, and ApoB levels were adjusted for in model 3. A receiver operating characteristic (ROC) analysis was conducted for the WHtR to evaluate whether it can precisely discriminate for IR in patients with PCOS. All p-values were two-sided, and *p*<0.05 was considered statistically significant.

## 3 Results

We included 882 PCOSAct participants, of which 458 (51.9%) were diagnosed with IR. The mean ages (± standard deviation) were 28.05 (± 3.39) years and 27.84 (± 3.23) years for patients with IR and without IR, respectively (*p*=0.341).

### 3.1 Anthropometric and biochemical characteristics of the patients with PCOS

**Table 1** shows the anthropometric and biochemical characteristics of the patients with PCOS categorized by the existence of IR.

**Table 1:** Anthropometric and biochemical characteristics of the women with PCOS.

Characteristic	Insulin resistance		P value
	No (N=424)	Yes (N=458)	
Age (y)	27.84 ± 3.23	28.05 ± 3.39	0.341
BMI (kg/m^2^)	21.48 (4.15)	25.60 (5.21)	<0.001
WC (cm)	79.52 ± 8.81	90.40 ± 10.84	<0.001
WHtR	0.50 ± 0.06	0.56 ± 0.07	<0.001
WHR	0.83 (0.08)	0.88 (0.09)	<0.001
SBP (mmHg)	110 (15)	113 (10)	<0.001
DBP (mmHg)	72 (10)	75 (10)	<0.001
Estradiol (pmol/L)	203.50 (112.50)	193.75 (96.50)	0.015
Progesterone (ng/ml)	1.76 (1.17) [423]	1.72 (1.25) [455]	0.207
FSH (mIU/mL)	6.17 ± 1.61 [423]	5.96 ± 1.70	0.058
LH (mIU/mL)	10.30 (9.46) [423]	9.00 (7.03) [457]	<0.001
LH/FSH ratio	1.75 (1.43) [423]	1.47 (1.06) [457]	<0.001
Total testosterone (nmol/L)	1.65 ± 0.61	1.67 ± 0.67	0.572
Free testosterone (pg/ml)	2.23 ± 0.88 [418]	2.32 ± 0.79 [452]	0.130
Free androgen Index	3.36 (3.68) [419]	6.09 (5.90) [455]	<0.001
Sex hormone binding globulin (nmol/L)	47.40 (36.40) [419]	24.90 (20.00) [455]	<0.001
Fasting glucose (mmol/L)	4.72 (0.89)	5.36 (0.92)	<0.001
Fasting insulin (µIU/mL)	7.59 (4.56)	18.62 (11.85)	<0.001
HOMA-IR	1.58 (1.00)	4.23 (2.99)	<0.001
Cholesterol (mmol/L)	4.53 ± 0.96	4.94 ± 1.15	<0.001
Triglyceride (mmol/L)	1.03 (0.63)	1.72 (1.25)	<0.001
High-density lipoprotein (mmol/L)	1.34 ± 0.38	1.21 ± 0.35	<0.001
Low-density lipoprotein (mmol/L)	2.81 ± 0.76	3.12 ± 0.92 [457]	<0.001
Lipoprotein A (mg/L)	100.70 (83.70) [423]	102.05 (87.0) [456]	0.888
Apolipoprotein A1 (g/L)	1.51 ± 0.32	1.51 ± 0.31	0.857
Apolipoprotein B (g/L)	0.80 ± 0.30	0.99 ± 0.30 [457]	<0.001

Values are expressed as mean ± standard deviation or median (interquartile range) unless stated otherwise. BMI, body mass index; WC, waist circumference; WHtR, waist-to-height ratio; WHR, waist-to-hip ratio; SBP, systolic blood pressure; DBP, diastolic blood pressure; FSH, follicle-stimulating hormone; LH, luteinizing hormone; HOMA, homeostasis model assessment; IR, insulin resistance.

The patients with PCOS and IR exhibited significantly higher anthropometric and metabolic parameters than those without IR. They displayed increased BMI (*p*<0.001), WC (*p*<0.001), WHtR (*p*<0.001), WHR (*p*<0.001), SBP (*p*<0.001), DBP (*p*<0.001), FAI (*p*<0.001), fasting glucose (*p*<0.001), fasting insulin (*p*<0.001), HOMA-IR (*p*<0.001), TC (*p*<0.001), TG (*p*<0.001), LDL-C (*p*<0.001), and ApoB (P<0.001) values but decreased E2 (*p*=0.015), LH (*p*<0.001), LH/FSH ratio (*p*<0.001), SHBG (*p*<0.001), and HDL-C (*p*<0.001) levels, respectively.

However, no significant differences were observed between the two groups in P (1.72[1.25] vs. 1.76[1.17]; *p*=0.207), FSH (5.96 ± 1.70 vs. 6.17 ± 1.61; *p*=0.058), TT (1.67 ± 0.67 vs. 1.65 ± 0.61; *p*=0.572), FT (2.32 ± 0.79 vs. 2.23 ± 0.88; *p*=0.130), lipoprotein A (102.05[87.0] vs. 100.70[83.70]; *p*=0.888), and ApoA1 (1.51 ± 0.31 vs. 1.51 ± 0.32; *p*=0.857) levels, respectively.

### 3.2 Correlations of the WHtR with the anthropometric and HOMA-IR data

**Figure 1:**
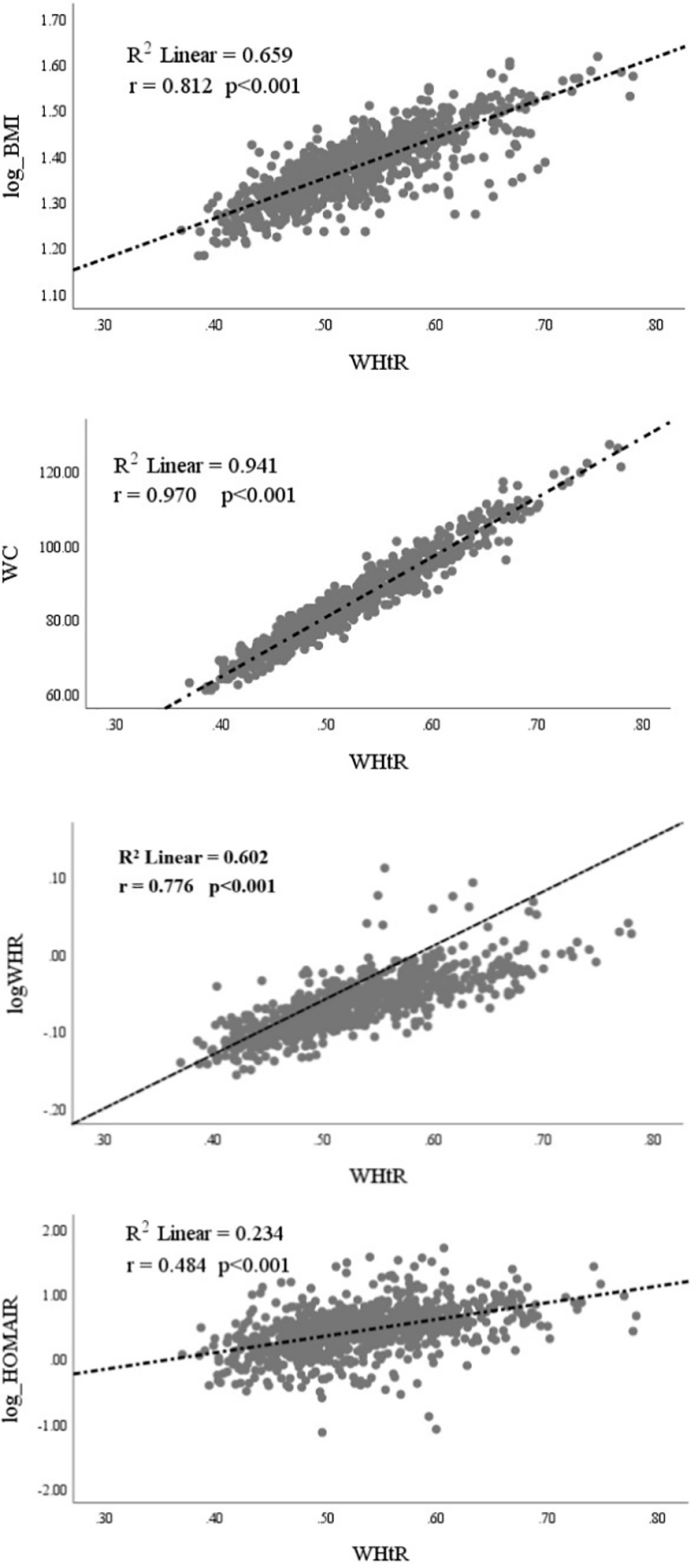
Correlation of Waist-to-Height Ratio (WHtR) with Log[Body Mass Index (BMI)], Waist Circumference (WC), Log[Waist-to-Hip Ratio (WHR)], and Log(HOMA-IR) for Insulin Resistance.

Pearson’s correlation analysis helped us to assess the correlations between the WHtR and BMI (log-transformed), WC, WHR (log-transformed), and HOMA-IR (log-transformed), as shown in **Figure 1**. The WHtR in women with PCOS was positively correlated with BMI (r=0.812; *p*<0.001), WC (r=0.970; *p*<0.001), WHR (r=0.776; *p*<0.001), and HOMA-IR (r=0.484; *p*<0.001).

In order to further explore the associations between the WHtR and HOMA-IR (log-transformed), multivariable linear regression analysis was used with potential confounding factor adjustments (**Table 2**). In model 1, without any adjustment, the WHtR was significantly associated with HOMA-IR, and the coefficient (95% CI) was 2.57 (2.26-2.88). In model 2 (with the adjustments for age, SBP, and DBP) and model 3 (with additional adjustments for E2, LH, LH/FSH ratio, FAI, SHBG, TC, TG, LDL-c, HDL-C, and ApoB), the WHtR was significantly associated with HOMA-IR. The resultant coefficients (95% CI) were 2.45 (2.13-2.77) and 1.98 (1.49-2.46), respectively.

**Table 2:** Association of the WHtR with IR in women with PCOS.

Linear regression on Log(IR)			
Variable	Coefficient (95% CI)	Standard coefficient	P value
Model 1, WHtR	2.57 (2.26-2.88)	0.484	<0.001
Model 2, WHtR	2.45 (2.13-2.77)	0.461	<0.001
Model 3, WHtR	1.98 (1.49-2.46)	0.271	<0.001
Logistic regression on IR			
Variable	OR	95% CI	P value
Model 1, WHtR×10	5.97	4.50 - 7.92	<0.001
Model 2, WHtR×10	5.69	4.27 - 7.60	<0.001
Model 3, WHtR×10	3.15	2.27 - 4.37	<0.001

Given the excessively large OR value observed for the WHtR in the logistic regression analysis on IR, the WHtR values were transformed by multiplying them by 10 to achieve accurate and interpretable OR values.

Model 1: no variable adjustment; Model 2: adjustments for age, SBP, and DBP; Model 3: adjustments for age, SBP, DBP, E2, LH, LH/FSH ratio, FAI, SHBG, TC, TG, LDL-C, HDL-C, and ApoB.

### 3.3 Association of the WHtR with IR

Additionally, multivariate logistic regression analysis helped us to identify the association between the WHtR and IR. As an excessively large OR value (>999.999) was observed for the WHtR in the IR logistic regression analysis, we multiplied the WHtR values by 10 to achieve precise and interpretable OR values. In model 1, without any confounding factor adjustment, the OR (95% CI) was 5.97 (4.50-7.92; *p*<0.001). In models 2 and 3, with the same adjustments as those in multivariate linear regression analysis, the association between WHtR (×10) and IR was statistically significant: the ORs (95% CI) were 5.69 (4.27-7.60; *p*<0.001) and 3.15 (2.27-4.37; *p*<0.001), respectively. This suggests that after adjusting for biomarkers relevant to IR, the risk of IR increased by 3.15 for every 0.1 unit increase in the WHtR.

**Figure 2:**
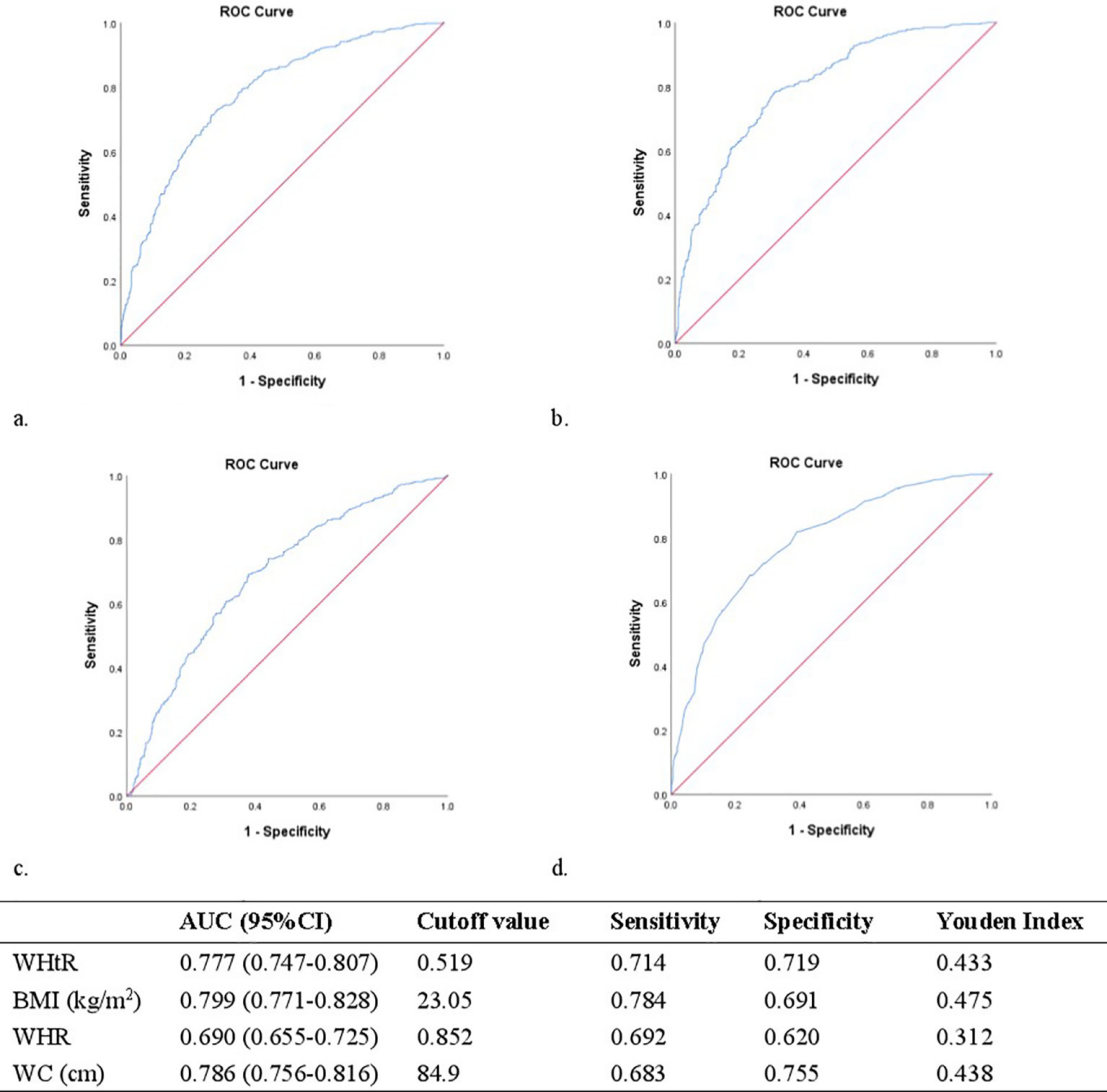
**(A)** Receiver operating characteristic curves and area under receiver operating characteristic curves for the detection of IR using WHtR. **(B)** Receiver operating characteristic curves and area under receiver operating characteristic curves for the detection of IR using BMI. **(C)** Receiver operating characteristic curves and area under receiver operating characteristic curves for the detection of IR using WHR. **(D)** Receiver operating characteristic curves and area under receiver operating characteristic curves for the detection of IR using WC.

ROC analysis helped us to determine the suggested WHtR cut-off values for IR. **Figure 2** shows that the WHtR performed significantly well in classifying women with PCOS as having IR. With an area under the ROC curve (AUC, 95% CI) of 0.690 (0.655-0.725; *p*<0.001), the WHtR outperformed the WHR. The AUC [95% CI] for the WHtR was 0.777 (0.747-0.807; *p*<0.001), comparable to BMI and WC, which had AUCs (95% CI) of 0.799 (0.771-0.828; *p*<0.001) and 0.786 (0.756-0.816; *p*<0.001), respectively. The best cut-off point for the WHtR in the patients with PCOS was 0.519 (Youden index = 0.433). The sensitivity, specificity, and cut-off values of the WHtR, BMI, WC, and WHR for IR prediction are listed in **Figure 2**.

Pearson’s correlation helped us to explore the association between several insulin resistance indexes, including fasting glucose, fasting insulin, and HOMA-IR, as well as anthropometric markers, including the WHtR, WC, and WHR (**Table 3**). Our results indicated that the WHtR exhibited similar correlations with fasting glucose, fasting insulin, and HOMA-IR, as well as WC and the WHR. Notably, the WHtR performed similarly to BMI and WC whereas the WHR was poorly associated when compared to the WHtR and WC. In the IR logistic regression analysis, the WHtR displayed a higher OR than BMI, WC, and WHR, suggesting that WHtR modifications have a greater impact on IR.

**Table 3:** Insulin resistance indexes and their associations with the WHtR, BMI, WC, and WHR.

Parameter	WHtR		BMI		WC		WHR	
	*r*	*p*	*r*	*p*	*r*	*p*	*r*	*p*
Fasting glucose	0.213	<0.001	0.205	<0.001	0.214	<0.001	0.128	<0.001
Fasting insulin	0.388	<0.001	0.394	<0.001	0.384	<0.001	0.281	<0.001
HOMA-IR	0.360	<0.001	0.356	<0.001	0.350	<0.001	0.261	<0.001
	WHtR×10		BMI		WC		WHR×10	
IR (OR 95%CI)*	5.97	4.50-7.92	1.39	1.32-1.46	1.12	1.10-1.14	2.42	1.94-3.01

Given the excessively large OR values for WHtR and WHR in IR logistic regression analysis, WHtR, and WHR values were transformed by multiplying them by 10 to achieve accurate and interpretable OR values.

*OR values were non-adjusted. Non-adjusted OR of WHtR (×10) for IR is shown in **Table 2**.

### 3.4 Comparison of IR condition based on the WHtR of patients with PCOS with a normal BMI, normal WC, or concurrent normal BMI and WC

As a previous study demonstrated that various IR predictors may vary among Chinese women with PCOS based on their body type (27), we conducted a comparison of IR conditions based on differing WHtRs in patients with PCOS with a normal BMI, or normal WC, or concurrent normal BMI and WC.

For patients with PCOS with a normal BMI, patients with IR were more likely to have a higher WHtR than those without IR (35.1% vs. 17.7%; *p*<0.001), and similar results were also found in patients with PCOS with a normal WC (16.6% vs. 7.5%; *p*=0.003) and patients with PCOS with concurrent normal BMI and WC (12.3% vs. 5.9%; *p*=0.043) (**Table 4**).

**Table 4:** Comparison of IR condition with different WHtRs in patients with PCOS with a normal BMI, normal WC, or concurrent normal BMI and WC.

	Low-WHtR	High-WHtR	*X^2^*	*P*
Normal BMI-PCOS				
IR			16.205	<0.001
Yes n (%)	96 (64.9)	52 (35.1)		
No n (%)	228 (82.3)	49 (17.7)		
Normal WC-PCOS				
IR			8.832	0.003
Yes n (%)	121 (83.4)	24 (16.6)		
No n (%)	296 (92.5)	24 (7.5)		
Normal BMI and WC-PCOS				
IR			4.081	0.043
Yes n (%)	93 (87.7)	13 (12.3)		
No n (%)	223 (94.1)	14 (5.9)		

Low-WHtR: WHtR ≤ 0.519; High-WHtR: WHtR>0.519; Normal BMI: 18.5≤BMI<24kg/m^2^; Normal WC: WC<85cm.

## 4 Discussion

We showed that the WHtR was independently associated with IR in patients with PCOS. In our study, we found that the prevalence rate of IR diagnosed using the HOMA-IR >2.6 was 51.9% in the patients with PCOS; this value was smaller than 74.9%, the prevalence assessed by hyperinsulinemic-euglycemic clamp (28). A 2013 study showed that the IR prevalence was lower in Chinese patients with PCOS (29).

The IR group’s levels of fasting glucose, fasting insulin, SBP, DBP, TC, TG, LDL-C, ApoB, and anthropometric indexes (BMI, WHtR, WHR, and WC) were significantly higher than the patients with PCOS but without IR. Moreover, a positive correlation was also observed between BMI, WHR, WC, HOMA-IR, and the WHtR. This was consistent with previous studies (30, 31). Furthermore, the WHtR was significantly associated with IR after potential confounding factor adjustment in multivariable linear regression and logistic regression analyses, respectively. Finally, ROC analysis demonstrated that when the WHtR became 0.519, it was very effective as an IR marker in patients with PCOS. Though IR develops independently from PCOS, it was found to induce the endocrine and reproductive traits of PCOS (32); this result was similar to our findings. In this study, women with PCOS and IR had lower values of E2 and SHBG as well as higher FAI levels. High androgen and low estrogen levels in PCOS are linked to infertility (33). However, we observed that patients with PCOS and IR had lower LH and LH/FSH ratios than those without IR. This is consistent with another observational Chinese study (34). Matthew et al. found a “metabolic” subtype of PCOS that was characterized by enhanced BMI, glucose, and insulin levels with lower SHBG and LH levels in an unsupervised, phenotypic clustering analysis with similar features as our IR-PCOS patients (35). Moreover, decreased LH concentration and LH/FSH ratio in overweight PCOS cases were more prevalent compared with non-overweight patients with PCOS. Thus, obesity can reduce LH pulse amplitude (36).

IR is significantly associated with all-cause and cardiovascular mortalities and is an important factor in regulating metabolic syndrome and diabetes (37, 38). Thus, its early identification is crucial for cardiovascular disease management in patients with PCOS. Currently, the most commonly used parameter for IR detection is the Homeostatic Model Assessment (HOMA). Other laboratory indexes include QUICKI, the Matsuda Index, and the Insulin Secretion-Sensitivity Index-2 (ISSI-2). All these have different values that vary for different ages, sexes, populations, and ethics (39). However, all these examinations require specialized equipment and trained professionals. In recent studies, anthropometric measurements such as BMI and WHR have been shown to be cost-effective tools for IR prediction and detection (40, 41). A novel anthropometric measurement, the WHtR, is an effective and precise anthropometric index to predict IR (42). Additionally, the WHtR was significantly associated with IR among anthropometric indicators in obese individuals (43). Another cross-sectional analysis of 2,231 participants with normal or impaired fasting glucose levels or type 2 diabetes indicated that the WHtR performed well in IR prediction, including in the subgroups that had impaired fasting glucose levels or type 2 diabetes, respectively (44). In our study, the WHtR was a powerful indicator for detecting IR in women with PCOS; the optimal cut-off point was 0.519 for patients with PCOS of reproductive age. Within the normal BMI populations, i.e., normal WC or normal BMI and WC, increased WHtR was significantly associated with IR, suggesting that the WHtR might identify IR earlier than BMI and WC, thereby enabling patients to adopt proactive preventive measures (**Table 4**). Unlike the BMI, the WHtR does not involve unit conversion and WC and height can be measured in the same unit. This makes the value more accurate. Furthermore, the WHtR varies between the sexes and among sexual maturation stages (45). However, studies on the WHtR and PCOS are inadequate, and additional studies are needed to prove the relationship in patients with PCOS and determine the exact cut-off points in different phenotypes. Notably, future comparisons between obese women with PCOS and obese women without PCOS could better illustrate the utility of WHtR in the PCOS population. Since the majority of our study participants were Han Chinese, variations in the height among individuals from diverse racial and ethnic backgrounds may limit our results’ applicability. Thus, future research endeavors should include extensive and diverse participants from various racial and ethnic backgrounds.

Our study had a few limitations. First, we diagnosed IR based on HOMA-IR instead of hyperinsulinemic-euglycemic clamp due to its complicated procedures, even though hyperinsulinemic-euglycemic clamp is the “gold standard” for IR assessment. Second, we could not provide an interpretation of the causality of associations due to our cross-sectional design. Third, a few social or lifestyle factors that might have influenced IR were not included as this was a secondary analysis based on PCOSAct. Finally, excluding the body composition analysis limited our results, thereby allowing the potential for further refinement.

## 5 Conclusion

In summary, early identification of IR is helpful for patients with PCOS to manage endocrine and metabolic disorders as well as reduce long-term health risks. This study showed that the WHtR was positively correlated with IR in PCOS-afflicted women. Thus, the WHtR could become a simple, practical, and reliable measure for identifying IR early in patients with PCOS.

## Data Availability

The original contributions presented in the study are included in the article/supplementary material. Further inquiries can be directed to the corresponding author.
